# Upper extremity sarcoma: impact of current practice guidelines and controversies on reconstructive approaches

**DOI:** 10.1051/sicotj/2017003

**Published:** 2017-02-21

**Authors:** Marek Dobke, Gina A. Mackert

**Affiliations:** 1 Division of Plastic Surgery, Department of Surgery, University of California San Diego San Diego CA 92103-8890 USA; 2 Department of Hand, Plastic and Reconstructive Surgery, Burn Center, BG Trauma Center 67071 Ludwigshafen Germany , the affiliated Department of ptastic surgery of the University of Heidelberg Germany

**Keywords:** Oncological upper extremity defect, Margins, Immediate reconstruction, Delayed reconstruction, Sarcoma

## Abstract

The goals of sarcoma management include both a cure and the functional preservation of involved tissues and adjacent critical structures with common opinions favoring immediate reconstruction. The question arises whether these goals are contradictory. This paper discusses the question based on the experience of 28 patients with different types of extremity sarcoma, with 24 surgically treated by the University of California San Diego (UCSD) orthopedic and plastic surgery team (2011–2016) and the collection of evidence from published practice guidelines, reviews, case studies, and clinical trials. Included are the impact of limb-sparing and functional reconstructive concepts, efforts regarding the adequacy of surgical margins, and the rationale of immediate versus delayed reconstructive approaches, and the disease-free status of sarcoma management.

## Introduction

To adequately address upper extremity sarcomas, and the ability to cure and functionally preserve the extremity, a multimodal and complex, function-conscientious management is often offered. The clinical evidence points out that surgical intervention and adequate excisional margins are the key elements to reducing morbidity and mortality as a consequence of treatment or disease [1–3]. While primary closure is often possible, it can frequently become problematic due to wound-healing complications in the setting of previously irradiated tissue and surgical wounds closed under tension. A reconstructive option of an immediate microsurgical free-tissue transfer for reconstructive purposes greatly extended the limits of limb salvage and the feasibility of immediate reconstruction [4, 5]. Consequently, limb-salvage surgery has become the standard of care for most soft tissue sarcomas. Primary bone malignancies, with the most common types being osteosarcoma, chondrosarcoma, and Ewing sarcoma, may require not only different diagnostic strategies and treatments, but also different reconstructive approaches with a frequent need for combined bone and soft tissue reconstruction [1, 4].

Functional results of immediate major reconstructive procedures can be impressive, and they are technically possible in an immediate single-stage setting; however, there are reports regarding errors in and problems with the diagnosis which precipitate a cautious and delayed approach to reconstruction. In these cases, where the diagnosis was not firmly established or confirmed, or where the emphasis was set on sparing the extremity instead of focusing on a long-term cure or patient survival under an established diagnosis, delaying definitive reconstruction should be thoroughly considered. A review of material referred to the multispecialty sarcoma treating center revealed 37% of the histopathological diagnoses change upon histopathological evaluation, including the grade of the sarcoma, and 82% of margins, previously declared as negative, return positive [6]. It is frequently not possible to collect the patient’s complete clinical information in a pre- or intraoperative setting, and the delay of reconstruction may be a prudent approach until all data has been collected. This delay may reduce the need for additional surgeries resulting from an inaccurate diagnosis or an uncertain situation regarding the margins [4–7]. Data, revealing a 25% chance of recurrence after wide excision of an extremity soft tissue sarcoma within five years after primary excision, reaffirm and underline the value of staged or delayed definitive reconstructive approaches [8]. Delayed reconstruction enables the completion of the margin analysis and the planning of the multimodal management of the extremity sarcoma [9].

For the multidisciplinary plastic and orthopedic surgical team, the prospect of the best functional outcome is not only related to the objective of anatomical extremity salvage, but also to the ability to perform complex and definitive anatomical and functional reconstruction. In other words, if performed without a certain diagnosis or clear margins, or in the event of a change in the clinical situation, the possibility that such a reconstruction might have to be revised or discarded in the future presents an argument for delayed reconstruction or at least for a customized approach with defect temporization until all necessary information and parameters are known.

Resecting sarcoma tumors to ensure negative surgical margins can be challenging. A full histopathological assessment including tumor grading may require time and may not be completed intraoperatively. A negative pressure vacuum-assisted wound closure (VAC) system sealing the defect for a few days after resection enables complete histopathological assessment of the margins prior to reconstruction, while also preserving acute surgical wound characteristics which enables a delay or staged reconstruction while still maintaining the advantages of immediate-like reconstruction. Some advocates of vacuum-assisted wound closure (VAC) devices for post-sarcoma-resection wound size reduction, granulation tissue induction for delayed or simple forms of repair, miss the by far most important VAC advantage: the fact that it can buy the necessary time for a full oncological wound assessment and the finalization of the reconstructive management planning. Using a VAC-system maintains not only acute wound biology, while retaining technical reconstructive options for immediate reconstruction but also enables the delivery of immediate brachytherapy followed by definitive reconstruction [10, 11].

On the other hand, there are clinical scenarios that necessitate immediate-definitive soft tissue reconstruction. Post-ablative or post-amputative defects may be so massive that some form of immediate reconstruction is mandatory. Sometimes post-amputative extremity parts may be utilized as donor tissue for a flap. In such instances, there is no other technical maneuver or choice other than to proceed with immediate reconstruction [10, 11]. Additionally, in cases of recurrent disease or in recurrent disease with margins not yet completely analyzed, or if other therapies (e.g., adjuvant radiation therapy) are planned, muscle flaps loaded with brachytherapy catheters can be a choice for reconstruction [2]. In fact, long-term prognostic models have not yet been developed and it is unknown to date how the combination and the sequencing of treatment modalities impacts the chance of a permanent cure [8, 13]. The goal of this study was to critically analyze the authors’ clinical material and evaluate whether the rationale regarding the choice for immediate versus delayed reconstruction was optimally balanced.

## Material and methods

The authors’ combined database includes 28 patients with advanced or complex upper extremity reconstruction defined as those necessitating multidisciplinary planning and surgical interventions (resection by the Orthopedic team and reconstruction by the Plastic Surgery team). The 24 patients (12 males and 12 females) were treated surgically between January 2011 and October 2016 with an average age of 45 years (range from 12 to 68 years). The tumor types within the study population are presented in Table 1. Four other patients (two with rhabdomyosarcoma and two with synovial-cell sarcoma) were evaluated and enrolled in neoadjuvant treatments succumbed to the disease before any surgery could take place.

**Table 1.** Histopathological diagnosis regarding the study population (surgical patients).

Type of tumor	Number of patients	Percent (%) of patients
Primary bone malignancies		
Osteosarcoma	4	17
Ewing sarcoma	2	8
Malignancies with major bone involvement		
Synovial-cell sarcoma	1	4
Liposarcoma	3	13
Hemorrhagic sarcoma	1	4
Soft tissue sarcomas without bone or marginal (periosteal) bone involvement		
Synovial-cell sarcoma	2	8
Malignant fibrous histiocytoma	2	8
Fibrosarcoma	2	8
Chondrosarcoma	2	8
Recurrent dermatofibroma protuberans displaying additional fibrosarcoma	2	8
Rhabdomyosarcoma	1	4
Epithelioid sarcoma	1	4
Myxoid sarcoma	1	4

Encrypted records of the multidisciplinary treatment planning conferences were reviewed blindly by two physicians (an orthopedic and a plastic surgeon) not involved in patient care. They analyzed individual patient age at diagnosis, sex, tumor site, type and duration of signs and symptoms, type of previous treatment (if any), initial diagnosis, local recurrences, regional or distant metastatic involvement, and the outcome as of November 2016. For each individual case, the treatment planning conference was re-enacted and factors determining the choice (timing) of post-resection reconstructive strategy were retrospectively analyzed. Specifically, utilizing the concept of decision analysis along a continuum regarding diagnostic certainty (no diagnosis, or diagnosis/information uncertain, or diagnosis established), and how predictable an intraoperative situation would be regarding the threshold for immediate reconstruction versus the need for further testing accompanied by delayed reconstruction with preparational wound temporization was determined (Table 2) [13–16].

**Table 2.** Factors influencing the choice of immediate-definitive extremity reconstruction.

Factors
Established diagnosis
Histological certainty about margins
Feasibility of performing major reconstruction at time of resection without high risk factors (i.e. anesthesia, infection, bleeding disorders, etc.)
Feasibility of conducting the procedure as a single-stage procedure
Absence of non-oncologic conditions contraindicating immediate reconstruction (e.g., infection)
Defect repair does not impair physical examination/surveillance
Completion of neoadjuvant treatment (if applicable)
Feasibility of completion of definitive reconstruction without delaying adjuvant treatment (if applicable)
Feasibility of combining reconstruction with adjuvant therapy if applicable (e.g. brachytherapy)
Primary size of tumor <5 cm
Patient age <50 years
Low tumor grading
New tumor recurrence
No presence/evidence of metastases

## Results

**Figure 1.**
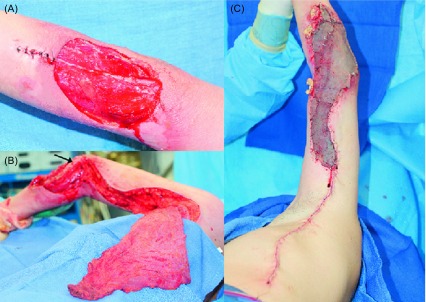
(A) Excised malignant fibrous histiocytoma requiring resection of soft tissue including the periosteum of the radial bone segment. Margins were negative and defined as wide [8, 9]. (B) Pedicled latissimus dorsi muscle flap prepared for functional muscle transfer: its humeral attachment and thoracodorsal neurovascular bundle were preserved. (C) The distal part of the flap was attached with through-and-through sutures to the proximal radial bone cortex and skin grafted for external coverage. Subsequent flap fixation to the bone (the site of musculodesis is marked by the arrow) allowed its use as a neo-elbow extending unit.

In eight cases (four of them with primary bone malignancies) resection was followed by immediate-definitive reconstruction, while in 16 cases the definitive procedure was delayed. Whenever possible during the original treatment planning, immediate-definitive reconstruction, concurrent with ablative or amputative procedures, was offered. Four of them were treated for recurrent disease. Retrospective analysis revealed that all of these patients were categorized as meeting the threshold points 100%. The follow-up (from one month to five years) revealed fatal progress of the disease in five cases (1. Osteosarcoma of the humeral bone after a forequarter amputation, 2. Pleomorphic malignant shoulder soft tissue fibrous histiocytoma, 3. elbow/proximal forearm area rhabdomyosarcoma, 4. Synovial-cell sarcoma of the soft tissue of the elbow area, 5. Liposarcoma of the arm), while the remaining patients appear to be disease free. All five patients underwent immediate reconstruction to cover critical defects, and to accommodate the placement of brachytherapy catheters within the flaps (shoulder sarcoma). One case entailed defect coverage and functional restoration of elbow extension in an elbow-area defect (Figure 1) [17]. The former was the only case where the design of the adjuvant modality impacted the reconstructive solution (the surveillance of the wound site would have been easier if the defect had been covered with a skin graft, which would have been technically possible).

Retroactive re-review of all cases revealed that, based on information at the time of original treatment planning (as opposed to an analysis during conferences on “Morbidity and Mortality”, with the previously lacking information known upon conclusion of the treatment), plans would be affirmed.

Of the 16 cases that underwent delayed reconstruction, 12 had been offered delayed reconstruction because of diagnostic uncertainty (diagnosis *per se* or non-established clear or safe margins) during the original pre-surgery treatment planning conference. In three cases, delayed reconstruction was performed because the diseased area was infected. The remaining case was offered delayed reconstruction because of the need for tumor grading and determination of how to complete the reconstruction (ultimately the patient underwent amputative surgery due to the suspected progression to a high-grade undifferentiated liposarcoma).

In the four cases with primary humeral bone involvement (two with Ewing sarcoma of the bone and two with osteosarcoma), segmental cortical bone resection was performed with autologous iliac crest bone grafts (Table 1). No segmental intercalary reconstruction was needed. In presented material, there were no major soft tissue defects associated with resection of primary bone sarcomas, which could require composite flaps, soft tissue wounds were repaired primarily.

## Discussion

Surgical guidelines for the treatment of upper extremity sarcomas evolved from amputative approaches to limb-sparing designs, with – if possible – immediate functional restoration procedures with neo- and adjuvant multimodal complementary treatments. Although, the lowest rate of recurrence is found in patients with amputative or compartmental (radical) resections, wide radical excisional surgery may result in a significant loss of tissue, especially if the tumor is large in diameter, crosses fascial planes, and/or invades neurovascular structures of extremities without survival benefit [1, 12–14]. On the other hand, conservative excisions with marginal margins may result in tumor rupture or local tissue contamination with tumor cells [1, 2, 15].

Safe surgical margins are a condition *sine qua non* for optimal long-term results. The definition “how much margin is safe” evolved on the basis of a better understanding of the biology of malignancies, utilizing the staging and grading systems while planning the management. As the expansion of available management options for advanced extremity malignancies, both surgical, including rehabilitative, and non-surgical therapeutic modalities, has increased the complexity of clinical decision making and is becoming more complex requiring specialized, multidisciplinary teams [1, 2, 8, 9, 13, 14].

**Figure 2.**
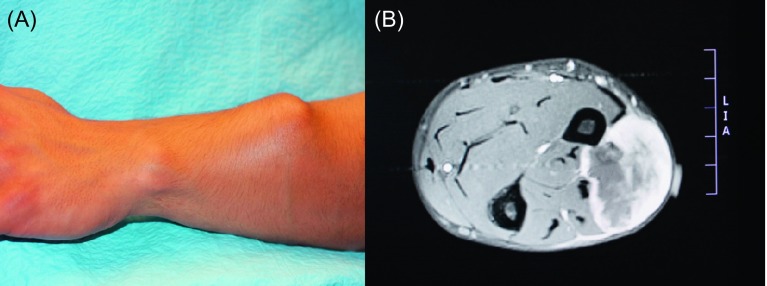
Twenty-three-year-old male with extraosseous rhabdomyosarcoma. The patient was being prepared for induction chemotherapy. The use of neoadjuvant chemotherapy was unanimously preferred by both medical and surgical members of the multidisciplinary team. The decision regarding the type of reconstruction was deferred and made dependent on the response to the neoadjuvant therapy. The patient succumbed to the disease while in therapy.

**Figure 3.**
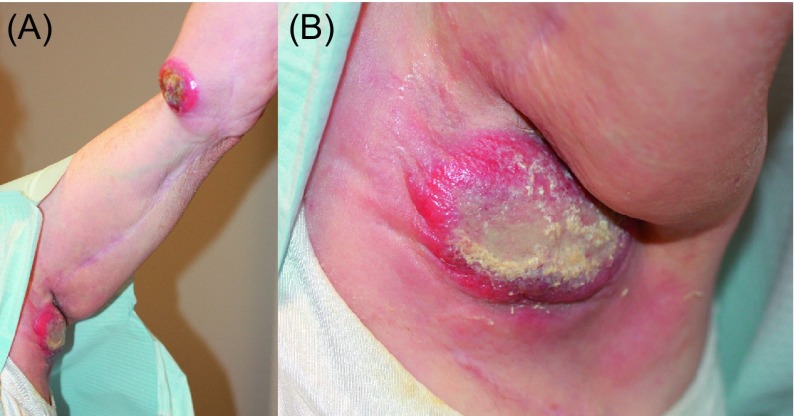
Aggressive tumors such as this synovial-cell sarcoma, stage G3T2bN1M1, require very invasive surgery and a customized reconstructive solution (in this case radical forequarter amputation with chest wall resection with a myocutaneous latissimus dorsi flap was planned). Neoadjuvant therapy was scheduled prior to surgery, however, the patient succumbed to the disease secondary to pulmonary metastases.

Surgeon consensus regarding the overall management plan for the group of patients analyzed in this study was 100%. Professional specialty and experience influences the treatment sequence preference with radiation oncologists, and practitioners experienced in sarcoma management lean toward preoperative neoadjuvant therapy while surgical intervention itself is not questioned (Figures 2 and 3) [14]. Usually the assessment of the margins after tumor removal surgery is the more problematic part. Difficulties with the determination of the margins heavily impact reconstructive surgeons as, in general, it is agreed that reconstructions, especially complex reconstructive approaches, should be definitive and should be avoided if margins are uncertain [2, 9]. For example, among the 117 patients who underwent sarcoma resection, margins were defined as intralesional in eight cases, marginal in 43, and wide in 66 patients by the treating team. Independent reviewers agreed only 71% with the original classification [9]. The most frequent disagreement was when addressing the distinction between marginal margins and wide margins [9].

The pivotal element of reconstructive planning, with the ability to be controlled by the patient and the surgeons, are surgical margins and the commitment to immediate or delayed definitive reconstruction. Concerns about false negative margins and the general difficulty in intraoperatively assessing large, three-dimensional fields, confirm the reasoning regarding the delay of complex flap reconstruction or a staged approach. Immediate flap reconstruction and anatomical tissue rearrangement during the reconstruction may make secondary tumor bed re-excision – if indicated – very challenging. Therefore, it should not be a surprise that the majority of reconstructive dilemmas evolved from uncertain disease diagnoses, to margins, or issues regarding the timing of the reconstruction [2, 16]. One cannot always be 100% sure about the margins, especially in cases of aggressive tumors that are difficult to trace with non-appositional growth such as in liposarcoma, synovial sarcoma, or angiosarcoma. Tissue mapping with serial biopsies of seemingly disease-free tissue in poorly differentiated or high risk tumors and quality imaging should always be considered prior to the reconstruction of soft tissues [18–20].

New imaging techniques may be useful in avoiding “one-size-fits all” management strategies, determining safe margins, and evaluating the responses to neo adjuvant and adjuvant treatments. 18F-fluorodeoxyglucose positron emission tomography/computed tomography (PET/CT) is a recently introduced tool that is helpful in mapping sarcoma affected tissues [18–20]. Modern three-dimensional precise tumor imaging techniques allow for relatively effective implementation of curative resections, which are both extensive and selective at the same time. Modern imaging allows the adoption and utilization of surgical and reconstructive approaches that are based on the recognition of biological barriers surrounding the tumor (e.g., fascia, periosteum, cartilage).

**Figure 4.**
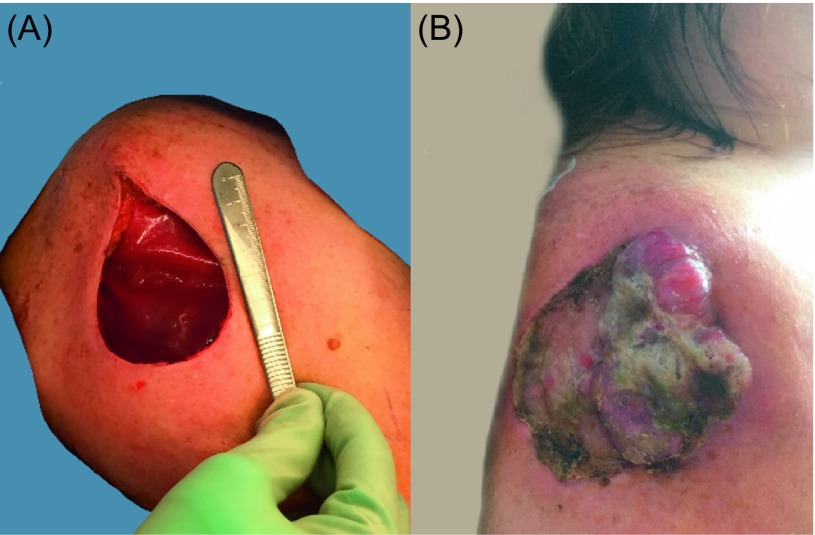
Liposarcoma of the right arm. (A) Wound after excision. (B) Recurrence a few weeks later. The majority of the already healed skin graft is damaged by the recurrent growth.

One case very vividly demonstrated the importance of being cautious and yet failing. The defect in the patient’s right arm after wide excision of the tumor with histologically clear margins was covered with Integra (Integra LifeSciences, Plainsboro, NJ), a temporary wound coverage, while awaiting the results of tumor grading (margins were clear but the tumor turned out to be of high grade) (Figure 4A). A VAC-system was placed over the Integra wound coverage. Four days after the excision, when the “histological negativity” of margins was reconfirmed, the patient underwent placement of a split thickness skin graft without further re-excision. The graft initially healed, two weeks later, however (four weeks after the initial excision), the patient developed a local recurrence and succumbed to metastatic disease a few months later (Figure 4B) [5, 15]. Most likely, false-negative margins or inadvertent tumor contamination of the wound during resection were the underlying causes leading to the consequences and the outcome observed in this case, which is depicted in Figure 4 [15].

Definitive closure may entail complicated reconstructive procedures which may need to be revised or re-radiated in the setting of positive margins or recurrent disease. In fact, the management of such situations is not well defined. It is unclear which approach is optimal to further limb salvage in case of a relapse near a prosthesis, allograft, or under a complex [10, 21]. One way to avoid these potential disadvantages of “premature” definitive reconstructions is to stage the surgeries. First an initial excision is completed, with temporary dressing of the wound bed for several days while margins are analyzed. A VAC-system is a practical, easy-to-apply, temporary modality, even in large three-dimensional defects. This allows the time necessary for the completion of evaluation of the margins, while maintaining defect features of the acute wound and avoiding the technical disadvantages which would be faced in chronic, fibrosed wound situations should there be a substantial delay in the reconstruction. The final coverage is deferred one or more days following a complete examination of the resected specimen. Focal positive margins may then be accurately addressed by re-excising appropriate portions of the surgical bed. In the case of extensive positive margins, wide re-excision or other appropriate surgical procedures can be considered. Most importantly, the original tumor bed has not been disturbed through flap reconstruction, so the concern of tumor seeding is eliminated. VAC-system dressings can even incorporate brachytherapy catheters allowing immediate adjuvant radiation prior to flap surgery and while the evaluation of the margins is pending. It should be mentioned that from the reconstructive surgeon’s perspective, adjuvant radiotherapy is advantageous because it does not affect the tissue quality within the radiated field, which – if utilized as neoadjuvant therapy – could technically impact and compromise microvascular surgery based reconstruction [11, 17, 21–23]. On the other hand, although it is not the scope of this report to assess advantages of neoadjuvant chemo- or radiotherapy, it is known that the sequencing of these modalities frequently prior to surgery enables the assessment of tumor response and facilitates R0 surgical resection. As discussed above, clear margins allow immediate- or early definitive reconstructions [24].

**Figure 5.**
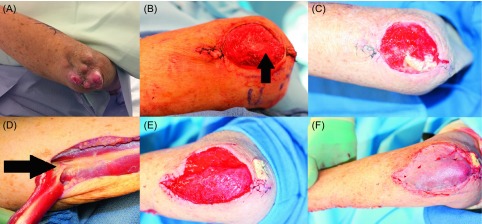
Seventy-one-year-old male with a myxoid sarcoma of the left elbow area. (A) Painless mass developing over the course of a few years with local progression and the development of satellite lesions. (B) Resection of the tumor including the periosteum (arrow) of the ulnar bone. As the examination of the margins was pending, this wound was temporarily protected by the subsequent application of a VAC-system. (C) One week after primary resection: the defect remains non-infected and prepared for reconstructive surgery. A muscle flap was necessary for sufficient coverage of the exposed bone and to provide well-vascularized coverage in case of adjuvant radiotherapy as part of the treatment protocol or in case of a recurrence. A skin graft would not have offered adequate durability attributes and would have been unlikely to heal over the periosteum-denuded bone. (D) Dissection of the flexor carpi ulnaris muscle flap: major vascular pedicle entering the deep surface of the muscle 6 cm distal to the elbow (arrow) with the visible delicate anastomoses forming the cubital vascular rete. (E) Defect coverage with the muscle flap. The distal tendon was embedded underneath the proximal wound edge and fixed percutaneously to a gauze bolster to ensure healing without flap dislodgment. (F) Split thickness skin graft covering the muscle flap surface.

Limb-sparing and defect reconstruction in the treatment of primary bone sarcomas or sarcomas grossly involving bone is sometimes an alternative to amputative surgery (Figure 3). For marginal bone involvement or bone exposure, principles of soft tissue reconstruction apply (Figures 1 and 5). For long bones, reconstruction with bone grafts to bridge relatively small bone defects and intercalary endoprostheses, allografts, vascularized bone, or the recycling of tumor-bearing bone for major or segmental defects are technical options. Microsurgical transfer of composite bone-soft-tissue flaps (e.g., fibula and overlying soft tissue) provides good long-term, durable solutions [25, 26].

In addition to functional objectives, reconstructive extremity surgery designs include the consideration of aesthetic aspects regarding the surface and the volumetric characteristics of a graft or a flap. For the correction of soft tissue contours, autologous fat transfer has been recently proposed as an option. Therefore, it is worthy to bring up some words of caution. A local recurrence of osteosarcoma, which occurred 13 years after the primary tumor removal, was reported following fat injections for contour improvement, with some evidence suggesting that fat grafts or progenitor cells may promote tumor growth [27].

## Conclusions

Relative concordance is notable between the decisions regarding immediate versus delayed definitive upper extremity reconstruction and the reconstructive technique chosen at the time of pre-resectional planning or intraoperatively. Appreciation of difficulties in establishing truly negative margins should lead to a customized and individualized approach for the reconstructive management of each patient. The recognition of the value of staged or delayed definitive reconstruction should be the *modus operandi* of the orthopedic-plastic surgery team treating advanced upper extremity malignancies.

## Conflicts of interest

MD and GAM certify that they have no financial conflict of interest (e.g., consultancies, stock ownership, equity interest, patent/licensing arrangements, etc.) in connection with this article.
